# Effect of fruit smoothie supplementation on psychological distress among people with substance use disorders receiving opioid agonist therapy: protocol for a randomised controlled trial (FruktBAR)

**DOI:** 10.1186/s40795-022-00582-z

**Published:** 2022-09-03

**Authors:** Lars T. Fadnes, Einar Furulund, Karl Trygve Druckrey-Fiskaaen, Tesfaye Madebo, Jørn Henrik Vold, Maria Olsvold, Marianne Cook Pierron, Siv-Elin Leirvåg Carlsen, Rune Blomhoff, Torgeir Gilje Lid, for the ATLAS4LAR Study Group, Vibeke Bråthen Buljovcic, Siv-Elin Leirvåg Carlsen, Jan Tore Daltveit, Karl Trygve Druckrey-Fiskaaen, Lars T. Fadnes, Trude Fondenes, Per Gundersen, Else-Marie Løberg, Mette Hegland Nordbotn, Maria Olsvold, Marianne Cook Pierron, Christine Sundal, Beate Haga Trettenes, Jørn-Henrik Vold, Maren Borsheim Bergsaker, Tine Selmer Cruickshank, Eivin Dahl, Tone Lise Eielsen, Torhild Fiskå, Einar Furulund, Eirik Holder, Torgeir Gilje Lid, Tesfaye Madebo, Ewa Joanna Wilk, Rune Blomhoff, Hege Berg Henriksen

**Affiliations:** 1 Bergen Addiction Research, Department of Addiction Medicine, Haukeland University Hospital, Bergen, Norway; 2 Department of Global Public Health and Primary Care, University of Bergen, Bergen, Norway; 3 Centre for Alcohol and Drug Research, Stavanger University Hospital, Stavanger, Norway; 4 Department of Respiratory Medicine, Stavanger University Hospital, Stavanger, Norway; 5 Division of Psychiatry, Haukeland University Hospital, Bergen, Norway; 6 Department of Nutrition, University of Oslo, Oslo, Norway; 7 Department of Clinical Service, Division of Cancer Medicine, Oslo University Hospital, Oslo, Norway; 8 Department of Public Health, University of Stavanger, Stavanger, Norway

**Keywords:** Fruit, Food supplementations, Opiate substitution treatment, Substance-related disorders

## Abstract

**Background:**

People with substance use disorders generally have unhealthy diets, including limited intake of fruit and vegetables. Evidence shows substantial health benefits from increasing fruit and vegetable consumption on various indicators and possibly also psychological distress. A pilot study has indicated that supplementation with fruit smoothie could be promising also among people receiving opioid agonist therapy for opioid dependence. *FruktBAR* will compare the efficacy of added fruit smoothie supplementation to people receiving opioid agonist therapy compared to standard treatment without added supplementation.

**Methods:**

*FruktBAR* is a multicentre, randomised controlled trial. The trial will aim to recruit 302 patients receiving opioid agonist therapy. The intervention involves daily supplementation with 250 ml fruit smoothie including a variety of fruits such as apple, pineapple, mango, bananas, orange, blueberries, passion fruit, coconut, lime, and blackcurrant. The main endpoints are 16 weeks after intervention initiation. Participants will be included and followed up during and after the intervention. The target group will be patients with opioid dependence receiving opioid agonist therapy from involved outpatient clinics in Bergen and Stavanger, two of the largest cities in Norway. The main outcome is psychological distress assessed with Hopkins Symptom Checklist (SCL-10) at the end of the intervention period 16 weeks after initiation, and will be compared between the intervention and control arms. Secondary outcome measures are changes in fatigue, physical functioning assessed with a 4-minute step-test, health-related quality of life, biochemical indicators of inflammation, and biochemical indicators of fruit intake.

**Discussion:**

This study will inform on the relative advantages or disadvantages of fruit supplementation in addition to the current medically and psychologically oriented treatment of people receiving opioid agonist therapy. If the supplementation is efficacious, it can be considered for further scale-up.

**Trial registration:**

Registered 2022-02-08 in ClinicalTrials.gov, identifier NCT05229770.

## Background

People with substance use disorders and particularly those with opioid dependence, have high morbidity, reduced quality of life, and a high risk of early mortality [1, 2]. Studies on people with opioid dependence in comparable settings have shown diet patterns with a low intake of fruits and vegetables, reflected in biochemical indicators [3, 4]. Folic acid and carotenoids are among the biochemical markers of intake of fruits and vegetables, and studies show correlations between these biochemical markers and longevity [5]. An inadequate intake of vegetables and fruits could be a risk factor for diseases and early deaths, and an increase in these could have beneficial effects on several health outcomes [5–7]. High consumption of fruits and vegetables is associated with a reduction in cardiovascular disease, cancers, and all-cause mortality.

In the Oslo Antioxidant study, a randomized controlled trial, people smoking tobacco received either kiwi fruits, a combination of various antioxidant-rich foods including fruit smoothies, or none except their habitual regular diet [8]. This study observed changed gene expressions linked with cellular protective mechanisms after an eight-week intervention period with kiwi or antioxidant-rich foods. Another randomized controlled trial among people with chronic obstructive lung disease in Greece showed less progression of lung diseases among those increasing the consumption of fruits and vegetables substantially [9]. Trials have also shown promising findings on psychological distress of healthy diets, including increasing fruits and vegetables in addition to other elements [10, 11]. The impact of fruit smoothie supplementation alone is uncertain, as its potential impact on inflammation [12].

Very few experimental studies focusing on nutrition have been conducted among people with substance use disorders. To our knowledge, there are no other trials having investigated the effects of fruits and vegetables on people with substance use disorders aiming to improve mental health and physical functioning. A recently conducted pilot study by our team with a similar intervention has shown favourable experiences [not yet published]. Thus, we will conduct a multicentre randomised controlled trial to test if daily intake of fruit smoothie over sixteen weeks could improve psychological well-being, physical function tests, and biochemical indicators.

## Objectives

This paper presents the protocol of the FruktBAR. The primary objective is to compare the effect of a daily supplement of 250 ml fruit smoothie on the level of psychological distress among people with substance use disorders receiving the OAT from outpatient clinics in Bergen and Stavanger (intervention arm), compared with standard treatment without supplement.

Secondary objectives are comparing the trial arms regarding physical function tests, fatigue, assessment of changes in quality of life, and biochemical indicators, including inflammation.

## Methods

### Study design

The study design of this study is a multicentre individually randomised controlled trial.

### Study settings and participants

The target group will be patients with opioid dependence receiving OAT from involved outpatient clinics in Bergen and Stavanger. Bergen and Stavanger have adopted an integrated treatment and care model for patients receiving OAT. In Bergen, OAT outpatient clinics are located in each district with follow-up of patients by health and social workers on a weekly basis with observed intakes of the OAT medications [2]. The OAT outpatient clinics are staffed by a consultant and a junior physician, in addition to nurses, psychologists, and social workers. This treatment model for people receiving OAT treatment provides a well-suited platform to test the integration of further interventions aiming to improve their health and life span.

### Eligibility criteria

Inclusion criteria for the trial are

- Receiving weekly OAT outpatient follow-up from an included outpatient clinic
- Having fruit and vegetable intake below five portions per day (assessed at screening)
- Giving informed consent

The following exclusion criteria will be used:

- Allergies or prior anaphylactic reactions involving fruits or vegetables
- Poorly regulated diabetes type 1 or 2 (HbA1c ≥54 mmol/mol)

### Interventions

Participants randomised to the intervention arm will receive a 250 ml fruit smoothie as a diet supplement for sixteen weeks. The fruit smoothies will be marketed products including combinations of the following fruits: apple, pineapple, mango, bananas, orange, blueberries, passion fruit, coconut, lime, and blackcurrant. The participants will receive a mixture of different smoothie types with the option of removing alternatives based on preferences. Fruit smoothie products will come in plastic bottles and will be delivered directly to the participants. Each participant will receive seven smoothie bottles per week with an oral agreement with each participant to consume one of these per day. Delivery of fruit smoothie will generally be given in parallel with the delivery of OAT medication. Participants randomised to standard treatment will receive regular OAT clinic follow-up without added supplementation.

### Outcomes

The primary outcome is psychological distress assessed with the Norwegian validated translation of the ten-item version of the Hopkins Symptom Checklist (SCL-10) in the mid of the intervention period 16 weeks after initiation (12–16) [13]. This will be evaluated with the mean SCL-10 item score and compared between the intervention and control arms.

Secondary outcomes are also measured at the same time 16 weeks after initiation and include the following:

- Changes in fatigue assessed with the Fatigue Symptom Scale (FSS-3) [14]

- Physical functioning assessed with a 4-minute step-test that measures the number of steps climbed in 4 minutes [15]

- Changes in health-related quality of life assessed with the five dimensions and five level EuroQoL scale (EQ-5D-5L), as well as a self-reported question on happiness with a 0 to 10 visual analogue scale [16]

- Change in biochemical indicators of inflammation (compared to baseline estimates)
  - All participants: Mean concentration of C-reactive protein in serum and total leukocyte count in blood
  - Randomized sub-group (*n* = 60, 1:1 intervention:control arm): IFN-gamma, IL-1beta, IL-1RA, IL-6, IL-8, IL-10, IL-17A, MCP-1, TNF-alfa measured in dried blood spots

- Biochemical indicators of fruit intake (compared to baseline estimates)
  - All participants: Folic acid levels
  - Randomized sub-group (*n* = 60, 1:1 intervention:control arm): carotenoids (vitamin A-related compounds) measured in dried blood spots

### Sample size

We calculated sample sizes for a two-sample means test based on the following assumptions:

- The power is set at 90% with a two-sided alpha (α) error of 5%.
- Mean SCL-10 score in the control arm is 2.2 (SD: 0.8). This is based on data from a similar cohort [17].
- Mean SCL-10 score in the intervention arm is 1.90 (SD: 0.8).
- Intervention:control ratio of 1:1.

Based on the above-mentioned assumptions, 302 persons are required (151 in the intervention arm and 151 persons in the control arm). Statistical power was calculated in Stata SE 17.0.

### Recruitment

All patients receiving OAT treatment from included clinics will be considered the reference target population. As part of an annual clinical assessment of patients receiving OAT linked to the ATLAS4LAR project [18], patients will receive information about the study and will be asked for consent to participate. All patients in the target population with inclusion and no exclusion criteria will be offered study participation until the targeted sample size has been reached. Participants giving informed consent will receive an extended clinical assessment, and be randomised for the study.

### Allocation and blinding

We will use a 1:1 randomisation ratio between the intervention and the control arm. Randomisation will be electronically registered. Blinding of patients is regarded as infeasible and patients will be informed about the follow-up they will receive. However, they will not receive information on follow-up in the other arm or the exact hypotheses for the study. The study will blind outcomes assessor.

### Data collection and management

**Fig. 1:**
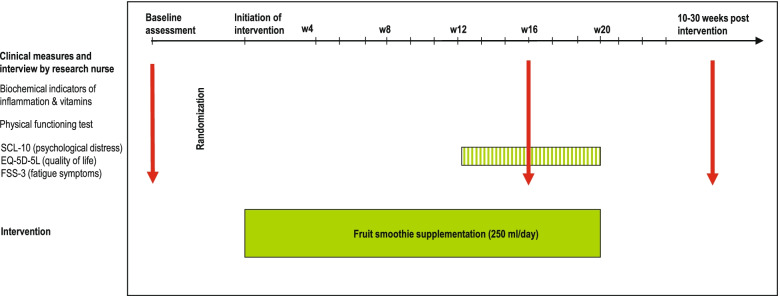
The figure gives an overview of the timing of the intervention and follow-up for the study. * The arrows are indications of when the various measurements are timed

Details on data collection and follow-up are given in Table 1*and* Fig. 1. Research nurses at the OAT clinics will measure/collect the primary outcome measures by blood samples and through interviews with all participants.

**Table 1:** Study flow chart presenting follow-up visits and assessments at each visit

	Screening (research nurse)	Treatment follow-up week 0 to 16 (nurses/social workers)	Intervention assessment week 16 (12–16 after initiation)	Intervention post-assessment (10–30 weeks after completion of intervention)
**Research nurse assessment**	X		X	X
- Informed consent	X			
- Eligibility assessment	X			
- Follow-up by OAT staff		X		
- Clinical assessment	X		X	
Biochemical tests	X		X	X
Physical funct. (4-min step-test)	X		X	X
Full blood count, ferritin etc	X		X	X
SCL-10 (mental health)	X		X	X
FSS-3 (fatigue symptoms)	X		X	X
EQ-5D-5L (quality of life)	X		X	X

### Analyses and statistical methods

A detailed analysis plan will be developed before data export and data analysis. Analysis methods will be conducted in line with the CONSORT and SPIRIT guidelines as far as possible [19–21]. Two-sided tests will be used, and *p* < 0.05 will be used as the statistical significance threshold. Descriptive tables for the participants will be presented with categorical variables that will be summarized with percentages, and for continuous variables as medians with interquartile ranges, or means with standard deviation for continuous variables with a Gaussian distribution. The main outcomes will be analysed with linear mixed models. Efficacy estimates of the outcomes will be presented with 95% confidence intervals. If there are substantial imbalances in potential confounders at baseline, estimates with adjustments for these will be presented in sensitivity analyses. For missing data, appropriate imputations based on pre-defined assumptions and analysis plan will be done when necessary.

### Potential harms and data monitoring

The participants receiving the intervention may experience inferior outcomes compared to the standard treatment. However, this is not considered as likely. Some might have allergies to components within the fruit smoothie, but severe allergies to these are rare, and people with severe allergies who are vulnerable to negative reactions will be excluded from participation in this trial. People with poorly regulated diabetes mellitus type 1 or 2 will be excluded from our trial as these might have needed treatment modifications. The *“therapeutic window”* of fruit smoothie is relatively wide, and except for some experiencing bloating and bowel distension, other side effects are uncommon.

All grade 3 and 4 adverse events are considered Serious Adverse Events (SAEs) and will be reported as such (including anaphylactic reactions). For safety evaluation, all SAEs occurring during the trial follow-up period will be recorded. All SAEs will be followed according to current treatment guidelines until resolution or until a stable clinical endpoint is reached. There is no independent data monitoring committee, but the study coordination unit will ensure protocol adherence, study quality, and ethical conduct. If severe allergic or anaphylactic reactions occur, these will be treated according to the standard protocol for allergic reactions used for anaphylactic reactions to vaccines and medications. A subsequent assessment will include total and fruit-specific immunoglobulins E to evaluate potential links between the intervention and the SAEs.

## Discussion

The research project will improve understanding of potential effects of an intervention increasing the daily intake of fruit smoothie over a period of sixteen weeks, and whether it could improve mental health, affect inflammation, and physical functioning. If found effective, this could potentially reduce the large morbidity and mortality among people with substance use disorders receiving OAT. There is a range of known interventions relevant to people with substance use disorders, including OAT and treatment of concurrent infections [22, 23]. If our trial is found effective, dietary interventions could potentially be integrated into a more comprehensive treatment model.

Our trial involves both a few limitations and several strengths. The trial is not fully blinded, although some masking measures are taken, including blinding of analysts, not informing patients on hypothesis, and using different people delivering the intervention and assessing the outcomes. The public funding of the study, ensures independency. The use of biological indicators that could be linked with the primary outcome reduces the risk of information biases. Further, the study is individually randomised, which minimizes potential confounding. The sample size of the study is sufficiently large to answer the primary objectives with a high degree of precision and is also assumed to have adequate precision for secondary objectives. Relating to safety, the trial is considered a low-risk study. Our trial design is less vulnerable to confounding from time trends than designs such as stepped-wedge designs.

If the fruit smoothie supplementation compared to standard care is efficacious in improving treatment outcomes, the intervention could have potential for scale-up.

## Data Availability

Trial outcome data is not yet available. For requests related to data, the corresponding author Lars T. Fadnes can be contacted (email: lars.fadnes@uib.no).

## Appendix

**Links with further information on fruit smoothie products:**

The smoothie products that will be used include mango and passion fruit: https://www.bama.no/produkter/smoothies/mango-og-pasjonsfrukt

Pineapple and coconut: https://www.bama.no/produkter/smoothies/ananas-og-kokos

Blueberries and apple: https://www.bama.no/produkter/smoothies/blabar-og-eple

Pineapple and mango: https://www.bama.no/produkter/smoothies/ananas-og-mango.

## Abbreviations

OAT: Opioid agonist therapy
SAE: Severe adverse effects
